# Synthesis of Extended Uridine Phosphonates Derived from an Allosteric P2Y_2_ Receptor Ligand

**DOI:** 10.3390/molecules19044313

**Published:** 2014-04-04

**Authors:** Lijun Song, Martijn D. P. Risseeuw, Izet Karalic, Matthew O. Barrett, Kyle A. Brown, T. Kendall Harden, Serge Van Calenbergh

**Affiliations:** 1 Laboratory for Medicinal Chemistry, Ghent University, Harelbekestraat 72, B-9000 Ghent, Belgium; E-Mails: lijun.song@ugent.be (L.S.); martijn.risseeuw@ugent.be (M.D.P.R.); izet.karalic@ugent.be (I.K.); 2 Department of Pharmacology, University of North Carolina, School of Medicine, Chapel Hill, NC 27599-7365, USA; E-Mails: matthew_barrett@med.unc.edu (M.O.B.); kyle_brown@med.unc.edu (K.A.B.); kendall_harden@med.unc.edu (T.K.H.)

**Keywords:** nucleoside phosphonates, extended uridine, P2Y_2_ receptor

## Abstract

In this study we report the synthesis of C5/C6-fused uridine phosphonates that are structurally related to earlier reported allosteric P2Y_2_ receptor ligands. A silyl-Hilbert-Johnson reaction of six quinazoline-2,4-(1*H*,3*H*)-dione-like base moieties with a suitable ribofuranosephosphonate afforded the desired analogues after full deprotection. In contrast to the parent 5-(4-fluoropheny)uridine phosphonate, the present extended-base uridine phosphonates essentially failed to modulate the P2Y_2_ receptor.

## 1. Introduction

Amongst the many physiological functions of nucleotides, their capacity to mediate signal transmission via cell-surface receptors was discovered in 1929 [1]. Purinergic receptors are subdivided into two families: metabotropic P2Y receptors (P2YRs) and fast acting P2X ion channels [2]. The P2X receptors generally respond to the native agonist ATP, whereas P2YRs can be activated by ATP, UTP, UDP and UDP-glucose. The P2YRs family consist of eight members, which can be divided in two subgroups based on receptor structure and second messenger system: P2Y_1,2,4,6,11_ and P2Y_12,13,14_ [3]. Ligand preferences of the eight P2YRs have been well established: P2Y_1_ (ADP), P2Y_2_ (UTP=ATP), P2Y_4_ (UTP), P2Y_6_ (UDP), P2Y_11_ (ATP, NAD^+^), P2Y_12 _ (ADP), P2Y_13_ (ADP), and P2Y_14_ (UDP, UDP nucleotide sugars) [4].

The P2Y_2_ receptor (P2Y_2_R) is responsible for many physiological functions and therefore attracts considerable attention for developing therapeutics. For example, the P2Y_2_R agonist diquafosol, which stimulates the secretion of chloride and water by epithelial cells, is approved in Japan for treatment of dry eye disease [5]. P2Y_2_R activation protects cardiomyocytes from hypoxia *in vitro* and reduces post-ischemic myocardial damage *in vivo* [2]. Furthermore, the P2Y_2_R is a promising target for the treatment of neurodegenerative diseases, including Alzheimer’s disease [6]. Recently, ATP activation of the P2Y_2_R was also shown to retard bone mineralization [1].

**Figure 1:**
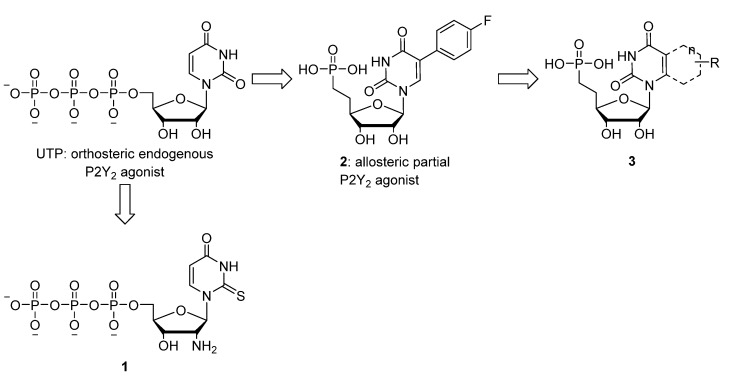
Structure of UTP, the selective P2Y_2_ agonist **1**, the allosteric partial agonist **2** and the target nucleoside phosphonates **3**.

The development of selective P2Y_2_R agonists and antagonists is a challenging task. Introduction of a 2'-amino and a 2-thio modification in UTP afforded analogue **1** (Figure 1) with enhanced potency and selectivity (versus P2Y_4_R) [7]. Unfortunately, most P2Y_2_R agonists contain a polyphosphate group, which is easily hydrolyzed by ecto-nucleotidases [4]. We recently reported a series of stable 5-substituted uridine phosphonate analogues, some of which (e.g., **2**, Figure 1) showed promising allosteric partial agonistic activity at P2Y_2_R [8]. Given the interesting profile of the 5-aryluridine analogues we envisaged modifying this nucleobase to a more rigid structure as a way to potentially enhance affinity for the P2Y_2_R. In this light we opted for a ring transformation from a 5-aryluracil to a quinazoline-2,4-dione motif, analogous to the biphenyl-naphthalene ring transformations often encountered in medicinal chemistry [9]. The first series of 5,6-annulated uridine analogues were based on the aromatic Topliss-scheme [6-H (3a), Cl (3c), OMe (3e) and Me (3d)] [10]. Additionally the 6-fluorine derivative **3b** was chosen based on the notable potency of parent compound **2** which bears a fluorine atom. Finally two nucleobases involving substitution of the benzene ring for a thiophene (**3f**) or a naphthalene core (**3g**) were included to gauge the effect of bioisosteric replacement or ring expansion of the nucleobase on the affinity for the P2Y_2_R.

## 2. Results and Discussion

### 2.1. Synthesis

To gain access to the target C_5_/C_6_-fused uridine phosphonates we first prepared the required “base” moieties **4a**–**g** by condensation of the appropriate *ortho*-amino aromatic carboxylic acids and urea at high temperature [11,12,13].

**Scheme 1:**
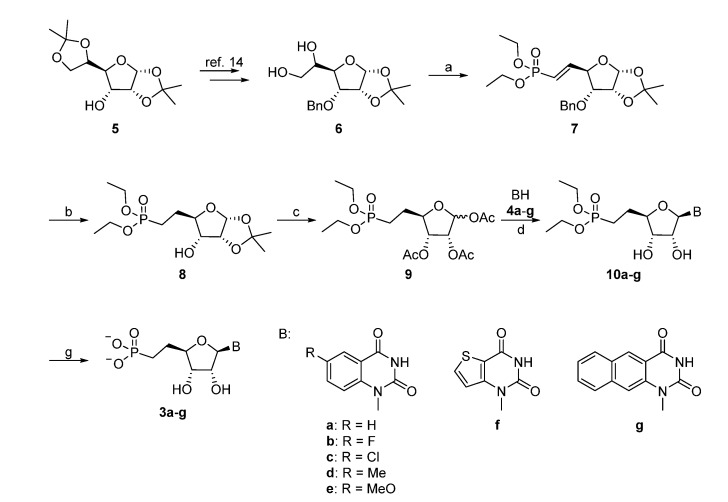
Synthesis of the Target Extended Uridine-5'-methylenephosphonates **3a**–**g**.

The synthesis of the appropriate glycosyl donor started from the known diol **6**, which was obtained in two steps from the commercial 1,2:5,6-*O*-diisopropylidene-α-d-allofuranose (**5**) following a known procedure (Scheme 1) [14]. Cleavage of the resulting vicinal diol with sodium periodate gave the aldehyde [15], which was used without purification in the subsequent Wittig-type reaction. During formation of the diethylvinylphosphonate **7**, C-4 may be prone to epimerization, which could be limited by performing the reaction at lower temperature. The yield for the conversion of **6** to **7** was significantly increased by using dichloromethane instead of ethanol for the periodate reaction. Hydrogenation with 10% Pd/C at atmospheric pressure allowed to simultaneous reduction of the double bond and removal of the benzyl group. One-pot deprotection of the isopropylidene group and acetylation of **8** afforded **9** (with an anomeric ratio of *ca.* 4:1 in favor of the α anomer), which was used in a silyl-Hilbert-Johnson reaction with the silylated benzo- or thieno[3,2-*d*]pyrimidine-2,4-diones to give compounds **10a**–**g** [16]. When HMDS was used as the silylating agent, careful removal of the silylating agents prior to the addition of the base and trimethylsilyltrifluoromethane sulfonate was required. Hence, the use of BSTFA for *in situ* silylation was preferred, despite the fact that it generally gave lower yields. Final deacetylation, followed by treatment with TMSBr/DCM afforded the desired nucleoside phosphonate analogues **3a**–**g**.

*Reagents and conditions*: (a) (i) NaIO_4_, DCM, H_2_O, rt; (ii) tetraethyl methylenediphosphonate, NaH, THF, 0 °C → rt; (b) H_2_, 10% Pd/C, rt; (c) (i) 60% AcOH, 85 °C, overnight; (ii) Ac_2_O, pyridine, overnight; (d) (i) BSTFA, ACN, 65 °C, 2 h; (ii) TMSOTf, CH_3_CN; (iii) NaOMe/MeOH, rt; (e) TMSBr, DCM, rt.

### 2.2. Pharmacological Evaluation

**Figure 2:**
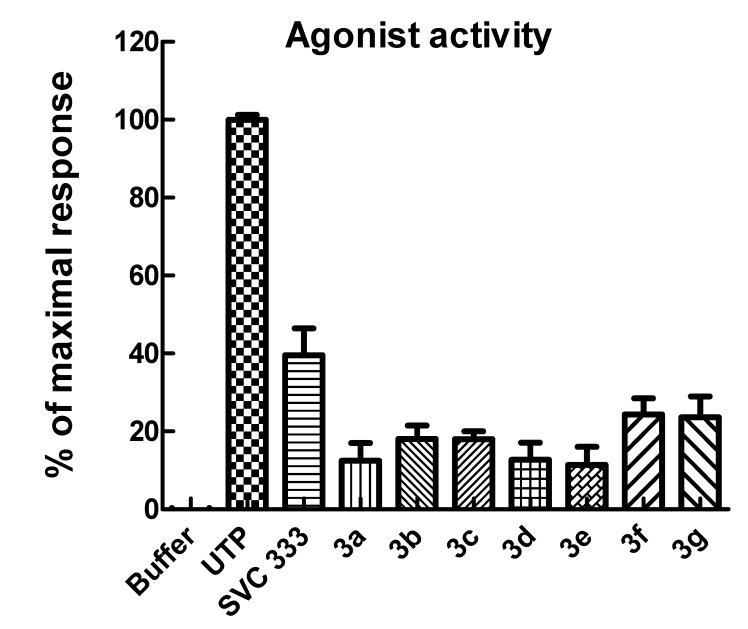
Inositol phosphate production in 1321N1 cells stably expressing the human P2Y_2_ receptor. After labeling with [^3^H]myo-inositol overnight, the cells were treated with agonists for 30 min at 37 °C in the presence of 10 mM LiCl, and inositol phosphate accumulation was quantified. The concentration of all phosphonate analagues was 30 μM and of UTP was 3 μM. The data are presented as the mean ± S.E.M. and are representative of results obtained in three separate experiments.

Agonist and antagonist activities of the C5/C6-fused uridine phosphonates at the P2Y_2_ receptor were determined measuring PLC-dependent phosphoinositide hydrolysis in 1321N1 human astrocytoma cells stably expressing the human P2Y_2_ receptor (1321N1-P2Y_2_ cells) [17,18] (Figure 2).

Compared to compound **2**, the current extended uridine phosphonates were found to stimulate the P2Y_2_ receptor to a lesser extent, indicating that rigidifying the base moiety is unfavorable for binding to a presumed allosteric binding site. It can be concluded that structural flexibility of the 5-substituent of the earlier discovered series of allosteric P2Y_2_ agonists is crucial for allosteric agonist activity.

**Figure 3:**
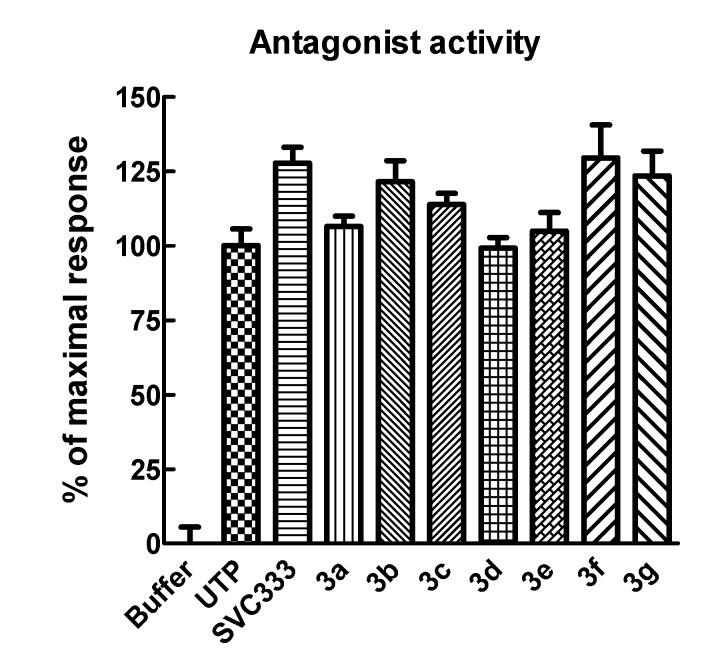
Effect of high concentrations of phosphonate analogues **3a**–**g** on the P2Y_2_ receptor-mediated activity of UTP. The effects of 30 μM concentrations of the indicated phosphonates were tested in the presence of 100 μM UTP in 1321N1-P2Y_2_ cells.

The possibility that the modified uridine 5'-phosphonate analogues could inhibit the effect of UTP by displacing it from the orthosteric site was examined by assessing their capacity at high concentrations to inhibit the effect of a near-maximal concentration of UTP (Figure 3). None of the phosphonate analogues gave an inhibitory effect on the action of UTP.

## 3. Experimental

### 3.1. General Methods and Materials

All reagents were from standard commercial sources and of analytic grade. All reaction were performed under argon atmosphere except specified otherwise. Dry solvents were directly acquired from commercial sources. Precoated Merck Silica Gel F254 plates were used for TLC. Spots were examined under ultraviolet light at 254 nm and further visualized by sulfuric acid-anisaldehyde spray. Column chromatography was performed on silica gel (200–400 mm, 60 Å). ^1^H, ^13^C, and ^31^P-NMR spectra were recorded in CDCl_3_, DMSO-*d*_6_, or D_2_O on a Varian Mercury 300 MHz spectrometer (Varian, Palo Alto, CA, USA). Chemical shifts are given in parts per million (ppm δ), δ relative to residual solvent peak or TMS for ^1^H and ^13^C and to external D_3_PO_4_ for ^31^P. Structural assignment was confirmed with COSY, HSQC and HMBC. Exact mass measurements were performed on a LCT Premier XE orthogonal time-of flight spectrometer with API-ES source (Waters, Zellik, Belgium) Waters LCT Premier XETM Time of flight (TOF) instrument. Samples were infused in a CH_3_CN/H_2_O (1:1) mixture at 10 mL/min.

*Diethyl ((E)-2-((3aR,5R,6R,6aR)-6-(benzyloxy)-2,2-dimethyltetrahydrofuro*[2,3-d][1,3]*dioxol-5-yl)-vinyl)phosphonate* (**7**): NaIO_4_ (0.52 g, 2.22 mmol) was added to a stirred solution of compound **6** in CH_2_Cl_2_ (15 mL) and H_2_O (10 mL) and the reaction mixture was stirred for 0.5 h at rt. The mixture was then filtrated and the filtrate was extracted with CH_2_Cl_2_. The combined organic layers were concentrated *in vacuo* to give the crude aldehyde, which was used in the next step without further purification. To a stirred suspension of NaH (0.31 g, 7.81 mmol) in dry THF (10 mL) was added tetraethyl methylene bisphosphonate (1.41 g, 4.88 mmol) under argon. After 0.5 h the mixture was cooled in an ice bath and a solution of the crude aldehyde in THF (10 mL) was added. The reaction mixture was allowed to warm to rt and stirred overnight. The crude mixture was diluted with water (20 mL) and extracted with ethyl acetate (3 × 20 mL). The combined organic layers were dried (Na_2_SO_4_) and concentrated *in vacuo*. Silica gel column chromatography (CH_2_Cl_2_/MeOH 97:3) afforded compound **7** as a colorless oil (0.77 g, 84.2%). ^1^H-NMR (300 MHz, CDCl_3_) δ: 1.28–1.35 (m, 6H, OCH_2_C*H*_3_), 1.36, 1.60 (2s, 6H, C(CH_3_)_2_), 3.53 (dd, *J = *9.2, 4.2 Hz, 1H, H-3'), 3.98–4.16 (m, 4H, OC*H*_2_CH_3_), 4.55–4.66 (m, 3H, H-2',H-4', OCH_2_Ph), 4.70–4.77 (m, 1H, OCH_2_Ph), 5.77 (d, *J = *3.8 Hz, 1H, H-1'), 5.96–6.12 (m, 1H, CH=CH), 6.74–6.90 (m, 1H, CH=CH), 7.28–7.42 (m, 5H, Ph). ^13^C-NMR (75 MHz, CDCl_3_) δ: 16.47, 16.56 (OCH_2_*C*H_3_), 26.63, 26.93 (C(*C*H_3_)_2_), 61.97 (t, *J = *4.7 Hz, O*C*H_2_CH_3_), 72.57 (O*C*H_2_Ph), 77.68 (d, *J = *9.4 Hz, C-4'), 78.04 (C-2'), 81.93 (d, *J = *1.7 Hz, C-3'), 104.15 (C-1'), 113.48 (C(CH_3_)_2_), 118.45 (d, *J* = 186.0 Hz, C-6'), 128.18 , 128.30, 128.68, 137.19 (Ph), 148.00 (d, *J = *6.1 Hz, C-5'). ^31^P-NMR (CDCl_3_) δ: 17.49. HRMS (ESI): Calculated for C_20_H_30_O_7_P [(M+H)^+^], 413.1724; found, 413.1754.

*Diethyl (2-((3aR,5R,6R,6aR)-6-hydroxy-2,2-dimethyltetrahydrofuro*[2,3-d][1,3]*dioxol-5-yl)ethyl) phosphonate* (**8**): To a solution of compound **7** (0.91 g, 2.22 mmol) in MeOH was added 10% Pd/C (0.5 g). The reaction mixture was stirred for 2 h under hydrogen atmosphere at rt and filtered. The filtrate was evaporated under vacuum and the residue purified by silica gel column chromatography (CH_2_Cl_2_/MeOH 97:3) to yield compound **8** as a colorless oil (0.58 g, 81.5%). ^1^H-NMR (300 MHz, CDCl_3_) δ: 1.29–1.35 (m, 6H, OCH_2_C*H*_3_), 1.36, 1.56 (2s, 6H, OC(CH_3_)_2_), 1.79–2.12 (m, 4H, H-5'a, H-5'b, H-6'a and H-6'b), 3.62 (ddd, *J = *10.3, 8.7, 5.3 Hz, 1H, H-3'), 3.77 (ddd, *J = *8.6, 6.7, 4.2 Hz, 1H, H-4'), 4.0–4.23 (m, 4H, OC*H*_2_CH_3_), 4.56 (dd, *J = *5.0, 4.1 Hz, 1H, H-2'), 5.77 (d, *J = *4.1 Hz, 1H, H-1); ^13^C-NMR (75 MHz, CDCl_3_) δ: 16.43, 16.50 (OCH_2_*C*H_3_), 20.61 (d, *J* = 141.7 Hz, C-6'), 24.99 (d, *J = *5.0 Hz, C-5'), 26.40, 26.52 (C(*C*H_3_)_2_), 61.67 (d, *J = *6.63 Hz, O*C*H_2_CH_3_), 75.55 (C-3'), 78.76 (C-2'), 79.49 (d, *J* = 15.8 Hz, C-4'), 103.65 (C-1'), 112.52 (*C*(CH_3_)_2_); ^31^P-NMR (CDCl_3_) δ: 31.66. HRMS (ESI): calculated for C_13_H_26_O_7_P [(M+H)^+^], 325.1411; found, 325.1429.

*(3R,4R,5R)-5-(2-(Diethoxyphosphoryl)ethyl)tetrahydrofuran-2,3,4-triyl triacetate* (**9**): A solution of compound **8** (0.45 g, 1.38 mmol) and 60% AcOH (10 mL) was heated at 80 °C under argon for 16 h. When the starting material was consumed completely, the reaction mixture was evaporated *in vacuo* and co-evaporated twice with AcCN. The residue was dissolved in dry pyridine (12 mL) at 0 °C under argon followed by dropwise addition of acetic anhydride (1.56 mL, 16.52 mmol). The reaction mixture was slowly warmed to rt and stirred overnight. Then it was concentrated and co-evaporated with toluene. The residue was purified by silica gel column chromatography (CH_2_Cl_2_/MeOH 95:5) to give **9 **(0.47 g, 83.2%) as a low melting solid (α:β anomeric ratio = 41:9). α anomer: ^1^H-NMR (300 MHz, CDCl_3_) δ: 1.22–1.30 (m, 6H, OCH_2_C*H*_3_), 1.63–1.94 (m, 4H, H-5'a, H-5′b, H-6'a and H-6'b), 1.99, 2.02, 2.04 (s, 9H, 3COCH_3_), 3.96–4.08 (m, 4H, OC*H*_2_CH_3_), 4.08–4.17 (m, 1H, H-4'), 5.09 (dd, *J = *7.0, 4.9 Hz, 1H, H-3'), 5.25 (d, *J = *4.9 Hz, 1H, H-2'), 6.06 (s, 1H, H-1'). ^13^C-NMR (75 MHz, CDCl_3_) δ: 16.49, 16.57 (OCH_2_CH_3_), 20.58, 20.60 (COCH_3_), 21.80 (d, *J* = 143.3 Hz, C-6'), 21.15 (CO*C*H_3_), 27.27 (d, *J* = 4.4 Hz, C-5'), 61.73, 61.82 (OCH_2_CH_3_), 73.27 (C-3'), 74.65 (C-2'), 81.27 (d, *J = *18.2 Hz, C-4'), 98.24 (C-1'), 169.19, 169.52, 169.87 (CO). ^31^P-NMR (CDCl_3_) δ: 30.82. β anomer: ^1^H-NMR (300 MHz, CDCl_3_) δ: 1.32 (t, *J = *7.0 Hz, 6H, OCH_2_CH_3_), 1.72–1.98 (m, 4H, H-5'a, H-5'b, H-6'a and H-6'b), 2.06, 2.10, 2.11 (s, 9H, 3 COCH_3_), 4.01–4.17 (m, 4H, OCH_2_CH_3_), 4.20–4.29 (m, 1H, H-4'), 5.02 (dd, *J = *6.8, 3.7 Hz, 1H, H-3'), 5.19 (dd, *J = *6.8, 4.6 Hz, 1H, H-2'), 6.36 (d, *J = *4.6 Hz, 1H, H-1'). ^13^C-NMR (75 MHz, CDCl_3_) δ: 16.54, 16.62 (OCH_2_CH_3_), 20.43, 20.76, 21.17 (CO*C*H_3_), 21.81 (d, *J* = 141.83 Hz, C-6'), 26.72 (d, *J = *4.4 Hz, C-5'), 61.81, 61.88 (OCH_2_CH_3_), 69.95 (C-2′), 72.02 (C-3'), 83.06 (d, *J = *17.1 Hz, C-4'), 93.95 (C-1'), 169.46, 169.80, 170.17 (CH_3_CO). ^31^P-NMR (CDCl_3_) δ: 30.57. ESI-HRMS for [C_16_H_27_O_10_P+H]^+^ calcd, 411.1420; found, 411.1436. HRMS (ESI): calculated for C_16_H_28_O_10_P [(M+H)^+^], 411.1415; found 411.1436.

### 3.2. General Procedures for the Silyl-Hilbert-Johnson Reaction to Synthesize Products **10a** to **10g**

Method 1: To a suspension of the appropriate quinazoline-2,4-(1*H*,3*H*)-dione (1.5 eq.) in dry acetonitrile (50 eq.) was added BSTFA (4 eq.) under argon. The solution was heated at 65 °C for 2 h. After cooling, a solution of triacetate **9** (1 eq.) in dry acetonitrile (50 eq.) and trimethylsilyl triﬂate (1.5 eq.) were added. The solution was stirred for 2 h at rt. The reaction was quenched with saturated aqueous NaHCO_3_ (50 mL for 1 mmol triacetate **9**) and extracted with dichloromethane (50 mL for 1 mmol triacetate **9**). The combined organic layer was washed with saturated aqueous NaHCO_3_ (3 × 50 mL for 1 mmol triacetate **9**), dried over anhydrous MgSO_4_ and evaporated. The crude mixture was dissolved in MeOH (50 eq.) and 5.4 N sodium methoxide (8 eq.) in MeOH was added dropwise. After 1 h, the mixture was neutralized with acetic acid (10 eq.) and evaporated. Purification of the residue by silica-gel column chromatography (CH_2_Cl_2_/MeOH 97:3→95:5) gave the pure product as a white foam.

Method 2: To a suspension of appropriate quinazoline-2,4-(1*H*,3*H*)-dione (1.5 eq.) in hexamethyl-disilazane (50 eq.) was added trimethylsilyl chloride (0.7 eq.) and pyridine (10 eq.) under argon. The mixture was stirred at 130 °C overnight. The reaction mixture was evaporated to dryness under high vacuum. To the obtained residue a solution of triacetate **9** (1 eq.) in dry acetonitrile (50 eq.) was added under nitrogen followed by trimethylsilyl triﬂate (1.5 eq.). The solution was stirred for 2 h at rt. and the work up was similar as described in method 1.

*Diethyl-(2-((2R,3S,4R,5R)-5-(2,4-dioxo-3,4-dihydroquinazolin-1(2H)-yl)-3,4-dihydroxy tetrahydrofuran-2-yl)ethyl)phosphonate* (**10a**): Following Method 1 reaction between **9** (0.47 g, 1.14 mmol) and **4a** (0.28 g, 1.71 mmol) yielded compound **10a** (0.22 g, 50%). ^1^H-NMR (300 MHz, CDCl_3_) δ: 1.27 (dt, *J = *18.3, 7.1 Hz, 6H, OCH_2_C*H*_3_), 1.79–2.15 (m, 4H, H-5'a, H-5'b, H-6'a and H-6'b), 2.15–2.36 (m), 4.45 (br s), 4.97 (br s, 2OH, NH), 3.88–3.97 (m, 1H, H-4'), 3.97–4.15 (m, 4H, OC*H*_2_CH_3_), 4.59 (t, *J = *6.7 Hz, 1H, H-3'), 4.80–4.89 (m, 1H, H-2'), 5.96 (d, *J = *2.0 Hz, 1H, H-1'), 7.19–7.27 (m, 1H), 7.45 (d, *J = *8.7 Hz, 1H), 7.59–7.67 (m, 1H), 8.14 (d, *J = *7.7 Hz, 1H, Ph). ^13^C-NMR (75 MHz, CDCl_3_) δ: 15.99–16.20 (m, OCH_2_*C*H_3_), 20.69 (d, *J* = 141.8 Hz, C-6'), 25.02 (d, *J = *4.4 Hz, C-5'), 61.66 (dd, *J = *12.2, 6.6 Hz, O*C*H_2_CH_3_), 71.95 (C-3'), 72.50 (C-2'), 82.32 (d, *J = *17.7 Hz, C-4'), 91.68 (C-1'), 114.27,115.92, 123.41, 128.37, 135.12 and 140.98 (Ph), 149.74 (C-2), 162.04 (C-4). ^31^P-NMR (CDCl_3_) δ: 32.08. HRMS (ESI): calculated for C_18_H_24_N_2_O_8_P [(M–H)^−^], 427.1276; found, 427.1284.

*Diethyl-(2-((2R,3S,4R,5R)-5-(6-fluoro-2,4-dioxo-3,4-dihydroquinazolin-1(2H)-yl)-3,4-dihydroxy-tetrahydrofuran-2-yl)ethyl)phosphonate* (**10b**): Following Method 2 reaction between **9** (0.34 g, 0.84 mmol) and **4b** (0.18 g, 1.01 mmol) yielded compound **10b** (133.3 mg, 61.3%). ^1^H-NMR (300 MHz, CDCl_3_) δ: 1.29 (dt, *J = *17.9, 7.1 Hz, 6H, OCH_2_CH_3_), 1.80–2.15 (m, 4H, H-5'a, H-5'b, H-6'a and H-6'b), 3.89–3.98 (m, 1H, H-4'), 3.98–4.16 (m, 4H, OCH_2_CH_3_), 4.29, 5.01, 10.31 (br s, 2OH, NH), 4.60 (t, *J = *6.2 Hz, 1H, H-3'), 4.83 (dd, *J = *6.2, 2.3 Hz, 1H, H-2'), 5.90 (d, *J = *2.6 Hz, 1H, H-1'), 7.33–7.42 (m, 1H, Ph), 7.43–7.50 (m, 1H, Ph), 7.78–7.85 (m,1H, Ph). ^13^C-NMR (75 MHz, CDCl_3_) δ: 16.57 (d, *J = *5.53 Hz, OCH_2_CH_3_), 21.14 (d, *J* = 140.1 Hz, C-6'), 25.52 (t, *J = *5.3 Hz, C-5'), 62.14 (dd, *J = *13.82, 6.6 Hz, OCH_2_CH_3_), 72.36 (C-3'), 73.01 (C-2'), 82.87 (d, *J = *17.1 Hz, C-4'), 92.40 (C-1'), 114.10–114.57 (m, Ph), 116.86 (d, *J = *7.2 Hz, Ph), 117.68–117.98 (m, Ph), 122.86–123.38 (m, Ph), 137.76 (Ph), 149.83 (C-2), 156.99–160.41 (m, Ph), 161.39 (C-4). ^31^P-NMR (CDCl_3_) δ: 32.71. HRMS (ESI): Calculated for C_18_H_23_FN_2_O_8_P [(M–H)^−^], 445.1182; found, 445.1188.

*Diethyl-(2-((2R,3S,4R,5R)-5-(6-chloro-2,4-dioxo-3,4-dihydroquinazolin-1(2H)-yl)-3,4-dihydroxytetra- hydrofuran-2-yl)ethyl)phosphonate* (**10c**): The reaction between **9** (0.34 g, 0.84 mmol) and **4c** (0.25 g, 1.26 mmol) as described for method 1 to yield compound **10c** (0.18 g, 45.1%). ^1^H-NMR (300 MHz, CDCl_3_) δ:1.28 (dt, *J = *16.69, 7.03 Hz, 6H, OCH_2_CH_3_), 1.79–2.14 (m, 4H, H-5'a, H-5'b, H-6'a and H-6'b), 3.92 (q, *J = *5.8 Hz, 1H, H-4'), 3.97–4.15 (m, 4H, OCH_2_CH_3_), 4.55 (t, *J = *6.7 Hz, 1H, H-3'), 4.85 (dd, *J = *6.15, 2.3 Hz, 1H, H-2'), 4.96, 10.49 (br s OH, NH), 5.89 (d, *J = *2.6 Hz, 1H, H-1'), 7.40 (d, *J = *9.4 Hz, 1H, Ph), 7.57 (dd, *J = *9.1, 2.6 Hz, 1H, Ph), 8.04 (d, *J = *2.3 Hz, 1H, Ph). ^13^C-NMR (75 MHz, CDCl_3_) δ: 16.44–16.63 (m, OCH_2_CH_3_), 21.13 (d, *J* = 141.0 Hz, C-6'), 25.48 (d, *J = *4.4 Hz, C-5'), 62.13 (dd, *J = *11.6, 6.6 Hz, OCH_2_CH_3_), 72.42 (C-3'), 72.80 (C-2'), 82.84 (d, *J = *17.1 Hz, C-4'), 92.25 (C-1'), 116.51, 117.55, 128.08, 129.76, 135.46 and 139.87 (Ph), 149.85 (C-2), 161.33 (C-4). ^31^P-NMR (CDCl_3_) δ: 32.68. HRMS (ESI): calculated for C_18_H_23_ClN_2_O_8_P [(M−H)^−^], 461.0886; found, 461.0899.

*Diethyl-(2-((2R,3S,4R,5R)-5-(6-methyl-2,4-dioxo-3,4-dihydroquinazolin-1(2H)-yl)-3,4-dihydroxytetra- hydrofuran-2-yl)ethyl)phosphonate* (**10d**): The reaction between **9** (0.25 g, 0.61 mmol) and **4d** (0.16 g, 0.91 mmol) as described for method 1 to yield compound **10d** (125.2 mg, 46.5%). ^1^H-NMR (300 MHz, CDCl_3_) δ:1.28 (dt, *J = *19.0, 7.1 Hz, 6H, OCH_2_CH_3_), 1.81–2.18 (m, 4H, H-5'a, H-5′b, H-6'a and H-6'b), 2.37 (s, 3H, CH_3_), 3.88–3.97 (m, 1H, H-4'), 3.97–4.16 (m, 4H, OCH_2_CH_3_), 4.28 (br s, 1H, OH), 4.56–4.68 (m, 1H, H-3'), 4.80–4.88 (m, 1H, H-2'), 4.98 (d, *J = *5.0 Hz, 1H, OH), 5.94 (d, *J = *2.3 Hz, 1H, H-1'), 7.34 (d, *J = *8.8 Hz, 1H, Ph), 7.40–7.48 (m, 1H, Ph), 7.94 (s, 1H, Ph), 10.28 (s, 1H, NH). ^13^C-NMR (75 MHz, CDCl_3_) δ: 15.78–16.15 (m, OCH_2_CH_3_), 20.51 (d, *J* = 141.0 Hz, C-6'), 19.95 (CH_3_), 24.83 (d, *J = *4.4 Hz, C-5'), 61.26–61.77 (m, OCH_2_CH_3_), 71.76 (C-3'), 72.55 (C-2'), 82.18 (d, *J = *17.1 Hz, C-4'), 91.52 (C-1'), 113.98, 115.56, 127.99, 133.17, 135.95 and 138.69 (Ph), 149.56 (C-2), 161.96 (C-4). ^31^P-NMR (CDCl_3_) δ: 32.84. HRMS (ESI): calculated for C_19_H_26_N_2_O_8_P [(M−H)^−^], 441.1432; found, 441.1427.

*Diethyl-(2-((2R,3S,4R,5R)-5-(6-methoxy-2,4-dioxo-3,4-dihydroquinazolin-1(2H)-yl)-3,4-dihydroxy- tetrahydrofuran-2-yl)ethyl)phosphonate* (**10e**): The reaction between **9** (0.25 g, 0.61 mmol) and **4e** (0.18 g, 0.91 mmol) as described for method 1 to yield compound **10e** (122.7 mg, 44.0%). ^1^H-NMR (300 MHz, CDCl_3_) δ:1.21–1.35 (m, 6H, OCH_2_CH_3_), 1.79–2.17 (m, 4H, H-5'a, H-5'b, H-6'a and H-6'b), 3.86 (s, 3H, OCH_3_), 3.89–3.97 (m, 1H, H-4'), 3.98–4.15 (m, 4H, OCH_2_CH_3_), 4.63 (t, *J = *6.7 Hz, 1H, H-3'), 4.85 (dd, *J = *6.0, 2.5 Hz, 1H, H-2'), 5.04 (br s, OH), 5.92 (d, *J = *2.3 Hz, 1H, H-1'), 7.20–7.25 (m, 1H, Ph), 7.39 (d, *J = *9.7 Hz, 1H, Ph), 7.59 (d, *J = *3.2 Hz, 1H, Ph), 10.27 (br s, 1H, NH). ^13^C-NMR (75 MHz, CDCl_3_) δ: 16.42–16.64 (m, OCH_2_CH_3_), 21.09 (d, *J* = 141.0 Hz, C-6'), 25.42 (d, *J = *4.4 Hz, C-5'), 56.02 (OCH_3_), 62.08 (dd, *J = *14.9, 6.6 Hz, OCH_2_CH_3_), 72.33 (C-3'), 73.08 (C-2'), 82.78 (d, *J = *17.1 Hz, C-4'), 92.19 (C-1'), 109.63, 116.27, 117.12, 124.35 and 135.41 (Ph), 149.94 (C-2), 155.95 (Ph), 162.30 (C-4). ^31^P-NMR (CDCl_3_) δ: 32.82. HRMS (ESI): calculated for C_19_H_26_N_2_O_9_P [(M−H)^−^], 457.1381; found, 457.1390.

*Diethyl-(2-((2R,3S,4R,5R)-5-(2,4-dioxo-3,4-dihydrothieno[3,2-d]pyrimidin-1(2H)-yl)-3,4-dihydroxy- tetrahydrofuran-2-yl)ethyl)phosphonate* (**10f**): The reaction between **9** (0.25 g, 0.61 mmol) and **4f** (0.15 g, 0.91 mmol) as described for method 1 to yield compound **10f** (198.8 mg, 75.2%). ^1^H-NMR (300 MHz, CDCl_3_) δ:1.20–1.35 (m, 6H, OCH_2_CH_3_), 1.79–2.15 (m, 4H, H-5'a, H-5'b, H-6'a and H-6'b), 3.92 (q, *J = *6.1 Hz, 1H, H-4'), 3.97–4.14 (m, 4H, OCH_2_CH_3_), 4.50 (t, *J = *6.3 Hz, 2H, H-3', OH), 4.80 (dd, *J = *5.9, 2.9 Hz, 1H, H-2'), 4.95 (br s, 1H, OH), 5.81 (d, *J = *3.2 Hz, 1H, H-1'), 7.13 (d, *J = *5.3 Hz, 1H, H-6), 7.73 (d, *J = *5.6 Hz, 1H, H-7), 10.33 (br s, 1H, NH). ^13^C-NMR (75 MHz, CDCl_3_) δ: 16.43–16.62 (m, OCH_2_CH_3_), 21.15 (d, *J* = 141.0 Hz, C-6'), 25.54 (d, *J = *4.4 Hz, C-5'), 62.12 (dd, *J = *14.4, 6.6 Hz, OCH_2_CH_3_), 72.39 (C-3'), 72.71 (C-2'), 83.14 (d, *J = *17.1 Hz, C-4'), 93.87 (C-1'), 114.38 (C-4a), 116.94 (C-6), 135.47 (C-7), 146.87 (C-7a), 151.09 (C-2), 158.53 (C-4). ^31^P-NMR (CDCl_3_) δ: 32.63. HRMS (ESI): calculated for C_16_H_22_N_2_O_8_PS [(M−H)^−^], 433.0840; found, 433.0838.

*Diethyl-(2-((2R,3S,4R,5R)-5-(2,4-dioxo-3,4-dihydrobenzo[g]quinazolin-1(2H)-yl)-3,4-dihydroxytetra- hydrofuran-2-yl)ethyl)phosphonate* (**10g**): The reaction between **9** (0.25 g, 0.61 mmol) and **4g** (0.19 g, 0.91 mmol) as described for method 1 to yield compound **10g** (125.0 mg, 43.1%). ^1^H-NMR (300 MHz, CDCl_3_) δ:1.22 (t, *J = *7.0 Hz, 6H, OCH_2_CH_3_), 1.77–2.07 (m, 4H, H-5'a, H-5'b, H-6'a and H-6'b), 3.81 (d, *J = *4.4 Hz, 1H, H-4'), 3.98 (quin, *J = *7.1 Hz, 4H, 2 × OCH_2_CH_3_), 4.13–4.26 (m, 1H, H-3'), 4.65 (br s, 1H, H-2'), 5.05 (d, *J = *6.2 Hz, 1H, OH), 5.21–5.32 (m, 1H, OH), 6.11 (d, *J = *4.1 Hz, 1H, H-1'), 7.54 (t, *J = *7.3 Hz, 1H, Ph), 7.66 (t, *J = *7.3 Hz, 1H, Ph), 7.91 (s, 1H, Ph), 8.06 (d, *J = *8.2 Hz, 1H, Ph), 8.14 (d, *J = *8.2 Hz, 1H, Ph), 8.75 (s, 1H, Ph), 11.68 (br s, 1H, NH). ^13^C-NMR (75 MHz, CDCl_3_) δ: 16.24, 16.31 (OCH_2_CH_3_), 20.74 (d, *J* = 140.1 Hz, C-6'), 25.63 (d, *J = *3.9 Hz, C-5'), 60.92 (dd, *J = *6.1, 2.8 Hz, OCH_2_CH_3_), 70.12 (C-2'), 72.01 (C-3'), 82.07 (d, *J = *17.1 Hz, C-4'), 91.20 (C-1'), 111.46, 116.35, 125.85, 127.59, 128.34, 129.05, 129.36, 129.52, 135.99 and 136.04 (Ph), 149.80 (C-2), 161.65 (C-4). ^31^P-NMR (CDCl_3_) δ: 32.08. HRMS (ESI): calculated for C_22_H_26_N_2_O_8_P [(M−H)^−^], 477.1432; found, 477.1419.

### 3.3. General Procedure for the Deprotection of **10a**–**g** to **3a**–**g**

The appropriate phosphonic ester **10** (1 eq.) was dissolved in dry CH_2_Cl_2_ (50 eq.) and treated with TMSBr (20 eq.) at 0 °C under argon. After stirring overnight at rt, the mixture was quenched with 7 N NH_3_/MeOH (20 eq.) and evaporated. The residue was dissolved in H_2_O (15 mL for 0.1 mmol phosphonic ester) and washed with CH_2_Cl_2_ (3 × 10 mL for 0.1 mmol phosphonic ester). The aqueous layer was evaporated and purified with RP high-performance liquid chromatography (HPLC, Phenomenex Luna C-18, H_2_O/0.1% HCOOH in CH_3_CN, 90:10→0:100 in 23 min, flow 17.5 mL/min) to give the desired product after lyophilization of the appropriate fractions.

*(2-((2R,3S,4R,5R)-5-(2,4-Dioxo-3,4-dihydroquinazolin-1(2H)-yl)-3,4-dihydroxytetrahydrofuran-2-yl)-ethyl) phosphonic acid* (**3a**): Deprotection of compound **10a** (0.13 g, 0.30 mmol) with TMSBr (0.91 g, 5.95 mmol) according to the general procedure yielded compound **3a** as a white power (32.2 mg, 29.1%). ^1^H-NMR (300 MHz, D_2_O) δ: 1.79–2.21 (m, 4H, H-5'a, H-5'b, H-6'a and H-6'b), 3.93–4.06 (m, 1H, H-4'), 4.34 (t, *J = *6.9 Hz, 1H, H-3'), 4.86 (dd, *J = *6.2, 4.4 Hz, 1H, H-2'), 6.09 (d, *J = *3.8 Hz, 1H, H-1'), 7.35 (t, *J = *7.5 Hz, 1H, Ph), 7.48 (d, *J = *8.5 Hz, 1H, Ph), 7.71–7.81 (m, 1H, Ph), 8.00 (d, *J = *7.9 Hz, 1H, Ph). ^13^C-NMR (75 MHz, D_2_O) δ: 22.89 (d, *J* = 135.8 Hz, C-6'), 25.82 (d, *J = *3.9 Hz, C-5'), 71.04 (C-2'), 72.22 (C-3'), 82.47 (d, *J = *17.7 Hz, C-4'), 90.85 (C-1'), 115.11, 115.72, 124.53, 128.05, 136.31 and 140.37 (Ph) 150.95 (C-2), 164.12 (C-4). ^31^P-NMR (D_2_O) δ: 29.02. HRMS (ESI): calculated for C_14_H_16_N_2_O_8_P [(M−H)^−^], 371.0650; found, 371.0651.

*(2-((2R,3S,4R,5R)-5-(6-fluoro-2,4-dioxo-3,4-dihydroquinazolin-1(2H)-yl)-3,4-dihydroxytetrahydro-furan-2-yl)ethyl)phosphonic acid* (**3b**): The reaction between compound **10b** (286.4 mg, 0.54 mmol) and TMSBr (1.65 g, 10.79 mmol) as described above, yielded compound **3b** as a white powder (15.7 mg, 14.8%). ^1^H-NMR (300 MHz, DMSO-d_6_) δ:1.50–2.04 (m, 4H, H-5'a, H-5'b, H-6'a and H-6'b), 3.65–3.76 (m, 1H, H-4'), 4.04 (t, *J = *6.7 Hz, 1H, H-3'), 4.49 (dd, *J = *6.4, 4.7 Hz, 1H, H-2'), 5.98 (d, *J = *4.4 Hz, 1H, H-1'), 6.66 (br s, 4H, 4OH), 7.47–7.55 (m, 1H, Ph), 7.55–7.64 (m, 1H, Ph), 7.71 (dd, *J = *8.2, 2.9 Hz, 1H, Ph), 11.79 (br s, 1H, NH). ^13^C-NMR (75 MHz, DMSO-d_6_) δ: 23.81 (d, *J* = 137.3 Hz, C-6'), 26.41 (C-5'), 69.88 (C-2'), 72.11 (C-3'), 82.49 (d, *J = *17.7 Hz, C-4'), 90.77 (C-1′'), 112.63–113.19 (m, C-5), 117.91 (d, *J = *7.7 Hz, C-4a), 118.12 (d, *J = *7.7 Hz, C-8), 122.31–122.85 (m, C-7), 136.87 (d, *J = *1.7 Hz, C-8a), 149.73 (C-2), 156.06–159.37 (m, C-6), 160.85 (d, *J = *2.2 Hz, C-4). ^31^P-NMR (CDCl_3_) δ: 26.92. HRMS (ESI): calculated for C_14_H_15_FN_2_O_8_P [(M−H)^−^], 389.0556; found, 389.0560.

*(2-((2R,3S,4R,5R)-5-(6-chloro-2,4-dioxo-3,4-dihydroquinazolin-1(2H)-yl)-3,4-dihydroxytetrahydro-furan-2-yl)ethyl)phosphonic acid* (**3c**): The reaction between compound **10c** (0.14 g, 0.31 mmol) and TMSBr (0.94 g, 6.11mmol) as described above, yielded compound **3c** as a white powder (49.0 mg, 39.5%). ^1^H-NMR (300 MHz, D_2_O) δ:1.80–2.17 (m, 4H, H-5'a, H-5'b, H-6'a and H-6'b), 3.87–3.98 (m, 1H, H-4'), 4.28 (t, *J = *7.0 Hz, 1H, H-3'), 4.76 (d, *J = *3.8 Hz, 1H, H-2'), 5.88 (d, *J = *3.8 Hz, 1H, H-1'), 7.25 (d, *J = *9.4 Hz, 1H, Ph), 7.50 (dd, *J = *9.1, 2.3 Hz, 1H, Ph), 7.56–7.61 (m, 1H, Ph). ^13^C-NMR (75 MHz, D_2_O) δ: 22.69 (d, *J* = 135.8 Hz, C-6'), 25.63 (d, *J = *3.3 Hz, C-5'), 71.29 (C-2'), 72.21 (C-3'), 82.29 (d, J = 18.3 Hz, C-4'), 90.98 (C-1'), 116.62, 116.96, 126.91, 129.32, 135.69 and 138.75 (Ph), 150.20 (C-2), 162.11 (C-4). ^31^P-NMR (D_2_O) δ: 30.04. HRMS (ESI): calculated for [C_14_H_15_ClN_2_O_8_P [(M−H)^−^], 405.0260; found, 405.0266.

(*2-((2R,3S,4R,5R)-3,4-dihydroxy-5-(6-methyl-2,4-dioxo-3,4-dihydroquinazolin-1(2H)-yl)tetrahydro-furan-2-yl)ethyl)phosphonic acid* (**3d**): The reaction between compound **10d** (0.11 g, 0.25 mmol) and TMSBr (0.77 g, 5.01mmol) as described above, yielded compound **3d** as a white powder (39.6 mg, 41.0%). ^1^H-NMR (300 MHz, D_2_O) δ:1.82–2.13 (m, 4H, H-5'a, H-5′b, H-6'a and H-6'b), 2.20 (s, 3H, CH_3_), 3.86–3.97 (m, 1H, H-4'), 4.28 (t, *J = *7.0 Hz, 1H, H-3'), 4.73 (m, 1H, H-2'), 5.86 (d, *J = *3.2 Hz, 1H, H-1'), 7.12 (d, *J = *8.5 Hz, 1H, Ph), 7.31–7.44 (m, 2H, Ph). ^13^C-NMR (75 MHz, D_2_O) δ: 19.62 (CH_3_), 22.76 (d, *J* = 133.5 Hz, C-6'), 25.66 (d, *J = *2.8 Hz, C-5′), 71.32 (C-2'), 72.26 (C-3'), 82.13 (d, *J = *17.7 Hz, C-4'), 90.82 (C-1′), 114.83, 114.96, 127.30, 134.51, 136.96 and 137.79 (Ph), 150.46 (C-2), 163.38 (C-4). ^31^P-NMR (D_2_O) δ: 29.86. HRMS (ESI): calculated for C_15_H_18_N_2_O_8_P [(M−H)^−^], 385.0806; found, 385.0819.

*(2-((2R,3S,4R,5R)-3,4-dihydroxy-5-(6-methoxy-2,4-dioxo-3,4-dihydroquinazolin-1(2H)-yl)tetrahydro-furan-2-yl)ethyl)phosphonic acid* (**3e**): The reaction between compound **10e** (0.11 g, 0.23 mmol) and TMSBr (0.70 g, 4.58 mmol) as described above, yielded compound **3e** as a white powder (48.9 mg, 53.1%). ^1^H-NMR (300 MHz, D_2_O) δ:1.78–2.18 (m, 4H, H-5'a, H-5'b, H-6'a and H-6'b), 3.86 (s, 3H, CH_3_), 3.93–4.01 (m, 1H, H-4'), 4.32 (t, *J* = 6.8 Hz, H-3'), 4.84 (dd, *J = *6.6, 4.2 Hz, 1H, H-2'), 6.04 (d, *J = *4.0 Hz, 1H, H-1'), 7.29–7.35 (m, 1H, Ph), 7.37–7.43 (m, 2H, Ph). ^13^C-NMR (75 MHz, D_2_O) δ: 22.99 (d, *J* = 126.1 Hz, C-6'), 25.51 (C-5'), 55.90 (CH_3_), 70.98 (C-2'), 72.21 (C-3'), 82.46 (d, *J = *18.2 Hz, C-4'), 90.83 (C-1'), 109.87, 116.65, 116.92, 123.80, 134.38 and 155.46 (Ph), 150.68 (C-2), 163.60 (C-4). ^31^P-NMR (D_2_O) δ: 28.81. HRMS (ESI): calculated for C_15_H_18_N_2_O_9_P [(M−H)^−^], 401.0755; found, 401.0755.

*(2-((2R,3S,4R,5R)-5-(2,4-dioxo-3,4-dihydrothieno*[3,2-d]*pyrimidin-1(2H)-yl)-3,4-dihydroxytetra- hydrofuran-2-yl)ethyl)phosphonic acid* (**3f**): The reaction between compound **10f** (0.18 g, 0.41 mmol) and TMSBr (1.24 g, 8.13 mmol) as described above, yielded compound **3f** as a white powder (75.6 mg, 49.2%). ^1^H-NMR (300 MHz, DMSO-*d*_6_) δ:1.48–2.04 (m, 4H, H-5'a, H-5'b, H-6'a and H-6'b), 3.67–3.77 (m, 1H, H-4'), 4.01 (t, *J = *6.4 Hz, 1H, H-3'), 4.41 (dd, *J = *6.4, 5.3 Hz, 1H, H-2'), 5.91 (d, *J = *5.0 Hz, 1H, H-1'), 6.53 (br s, 2H, 2OH), 7.25 (d, *J = *5.6 Hz, 1H, H-6), 8.12 (d, *J = *5.3 Hz, 1H, H-7), 11.62 (s, 1H, NH). ^13^C-NMR (75 MHz, DMSO-*d*_6_) δ: 23.78 (d, *J* = 135.8 Hz, C-6', 26.48 (C-5'), 70.41 (C-2'), 71.94 (C-3'), 83.00 (d, *J = *17.7 Hz, C-4'), 90.78 (C-1′), 113.85 (C-4a), 118.02 (C-6), 135.77 (C-7), 145.17 (C-7a), 150.77 (C-2), 157.91 (C-4). ^31^P-NMR (DMSO-*d*_6_) δ: 28.07. HRMS (ESI): calculated for C_12_H_14_N_2_O_8_PS [(M−H)^−^], 377.0214; found, 377.0197.

*(2-((2R,3S,4R,5R)-5-(2,4-dioxo-3,4-dihydrobenzo[g]quinazolin-1(2H)-yl)-3,4-dihydroxytetrahydro-furan-2-yl)ethyl)phosphonic acid* (**3g**): The reaction between compound **10g** (0.32 g, 0.56 mmol) and TMSBr (1.73 g, 11.30 mmol) as described above. The working up as follows: After stirring overnight, the reaction mixture was evaporated and co-distilled with toluene. The residue was dissolved with 7 N NH_3_/MeOH (10 mL) and stirred at room temperature for 3 h. The reaction mixture was concentrated and purified with RP high-performance liquid chromatography (HPLC, Phenomenex Luna C-18, H_2_O/0.1% HCOOH in CH_3_CN, 90:10→0:100 in 23 min, flow 17.5 mL/min) to give the compound **3g** as a white power (50.0 mg, 21.0%). ^1^H-NMR (300 MHz, DMSO-d_6_) δ:1.52–2.14 (m, 4H, H-5'a, H-5'b, H-6'a and H-6'b), 3.75–3.85 (m, 1H, H-4'), 4.16 (t, *J = *6.6 Hz, 1H, H-3'), 4.63 (dd, *J = *6.4, 4.1 Hz, 1H, H-2'), 5.21 (br s, 2H, 2OH), 6.10 (d, *J = *4.1 Hz, 1H, H-1'), 7.49–7.58 (m, 1H, Ph), 7.61–7.70 (m, 1H, Ph), 7.93 (s, 1H, Ph), 8.05 (d, *J = *8.2 Hz, 1H, Ph), 8.13 (d, *J = *8.2 Hz, 1H, Ph), 8.74 (s, 1H, Ph), 11.67 (s, 1H, NH). ^13^C-NMR (75 MHz, DMSO-d_6_) δ: 23.54 (d, *J* = 137.1 Hz, C-6'), 26.14–26.32 (m, C-5'), 69.83 (C-2'), 71.82 (C-3'), 82.23 (d, *J = *17.4 Hz, C-4'), 90.86 (C-1'), 111.25, 116.09, 125.56, 127.35, 128.06, 128.75, 129.11, 129.22, 135.70 and 135.74 (Ph), 149.54 (C-2), 161.38 (C-4). ^31^P-NMR (DMSO-d_6_) δ: 26.39. HRMS (ESI): calculated for C_18_H_18_N_2_O_9_P [(M−H)^−^], 421.0806; found, 421.0795.

### 3.4. Procedures for Phospholipase C assay

Stable cell lines for study of the human (h) P2Y_2_, were generated by retroviral expression of the receptor in 1321N1 human astrocytoma cells, which do not natively express P2YRs. Agonist-induced [^3^H]inositol phosphate production was measured in cells plated at 20,000 cells/well on 96-well plates two days prior to assay. Sixteen hours before the assay, the inositol lipid pool of the cells was radiolabeled by incubation in 100 μL of serum-free inositol-free Dulbecco’s modified Eagle’s medium, containing 1.0 μCi of [^3^H]myo-inositol. No changes of medium were made subsequent to the addition of [^3^H]inositol. On the day of the assay, cells were challenged with 25 μL of a five-fold concentrated solution of receptor agonists in 100 mM Hepes (*N*-(2-hydroxyethyl)-piperazine-N''-2-ethanesulfonic acid), pH 7.3 in HBSS, containing 50 mM LiCl for 30 min at 37 °C. Incubations were terminated by aspiration of the drug-containing medium and addition of 90 μL of ice-cold 50 mM formic acid. After 30 min, supernatants were neutralized with 30 μL of 150 mM NH_4_OH and applied to Dowex AG1-X8 anion exchange columns. Total inositol phosphates were eluted and radioactivity was measured using a liquid scintillation counter. Antagonist activities were conducted in a similar manner using a maximally efficatious dose of agonist with variable amount of antagonist.

## 4. Conclusions

Based on the earlier identification a 5-aryl-substituted uridine phosphonate analogue as a promising allosteric partial agonist of the P2Y_2_R, we prepared a small collection of a novel C_5_/C_6_-fused uridine phosphonates. Pharmacological evaluation revealed that the target compounds exhibit much weaker agonistic activity than the parent compound.
